# The efficacy of topical aminophylline in local fat reduction: A systematic review

**DOI:** 10.3389/fendo.2023.1087614

**Published:** 2023-02-16

**Authors:** Ramin Abdi Dezfouli, Ali Hosseinpour, Mostafa Qorbani, Elnaz Daneshzad

**Affiliations:** ^1^ Chronic Diseases Research Center, Endocrinology and Metabolism Population Sciences Institute, Tehran University of Medical Sciences, Tehran, Iran; ^2^ Non-Communicable Diseases Research Center, Alborz University of Medical Sciences, Karaj, Iran

**Keywords:** aminophylline, topical, fat reduction, thigh, lipolysis

## Abstract

**Background and aims:**

Some studies have reported that the topical forms with aminophylline as the active ingredient appear to be relatively effective on local fat burning while having no/minimal side effects. This systematic review accumulates all of the data on the local fat-burning potency of aminophylline topical formulation.

**Methods:**

Documents were retrieved from PubMed, Web of Science, and Scopus databases until Aug 2022. Data were extracted from clinical trials reporting the reduction in thigh or waist circumference as a result of using topical forms containing aminophylline. Screening of included studies was performed independently by two authors and the quality assessment of included studies was performed based on the Cochrane Collaboration’s approach.

**Results:**

Of the 802 initial studies, 5 studies were included in the systematic review. Several concentrations of aminophylline were used in different studies. Most studies administred the topical formulation on participants’ one thigh, and the other thigh was considered to be the control for comparing the fat reduction amount. Except for one study, all other studies reported that all participants lost more fat on the treated area than the control groups. The amount of fat reduction differed in studies regarding their different aminophylline concentrations and administration routines. In the case of side effects, except for some studies reporting skin rashes, other studies reported no significant side effects at all.

**Conclusions:**

Aminophylline topical formulation offers a safe, effective, and much less invasive alternative to cosmetic surgery for localized fat reduction. It seems that the 0.5% concentration, administered five times a week for five weeks is the most potent concentration. However, more high-quality clinical trials are needed to verify this conclusion.

**Systematic review registration:**

https://www.crd.york.ac.uk/prospero/, identifier CRD42022353578.

## Introduction

Obesity is a multifactorial chronic and progressive disease of excess adipose tissue that can occur at any age (1). According to reports (2), 46% of adults aged ≥20 are overweight or obese worldwide. Moreover, United States (US) has the highest obesity rate, with a prevalence of 34% in adults (3). Studies have also predicted that nearly 1.92 billion people worldwide will be obese or overweight by 2030 (2). On top of the physical health risks associated with obesity, negative psychological consequences are also inevitable. Depression (4–6), body image dissatisfaction (7), and stress (7–9) are all examples of this regard that can reduce one’s quality of life both directly and indirectly.

Regarding obesity management strategies, behavioral modification is one of the main approaches. However, depressed mood and anxiety reduce obese people’s functioning and their adherence to lifestyle changes (8). Medical interventions, on the other hand, are becoming more popular, and the obesity control guidelines strongly recommend medical interventions for overweight patients (10). In the case of these interventions, the most common approaches are oral/injectable anti-obesity medications (11–13) and bariatric surgeries (14). However, there are some drawbacks to these medical interventions too. The most significant disadvantages of oral anti-obesity agents (i.e., phentermine/topiramate, naltrexone/bupropion, and orlistat) are their systematic side effects, such as neuro-psychiatric, fetal, and cardiovascular side effects (11–13). Furthermore, it has been reported that the degree of weight loss provided by anti-obesity medications does not typically provide the type of cosmetic benefit that many patients seek (11, 12). In the case of injectable medications (i.e., liraglutide) and bariatric surgeries, their main disadvantage is their aggressive method of administration and their high costs (12, 14, 15). As a result, the most appropriate drug is one that is more effective, has fewer side effects, is less expensive, and is administered in a non-aggressive manner.

Topical formulations, on the other hand, are non-aggressive methods of drug administration and have significantly fewer side effects due to their low systematic absorbance. Regarding efficacy, several attempts have been made to develop a topical formulation for local fat loss (16–21). Among all, some studies have reported that the topical forms with aminophylline as the active ingredient appear to be relatively effective on local fat burning while having no/minimal side effects.

Therefore, the goal of this study was to accumulate all of the data on the local fat-burning potency of aminophylline topical formulation in order to get one step closer to developing topical and effective fat burner agents.

## Methods

Prior to the start of the study, a detailed research protocol was prepared and registered in the international prospective register of systematic reviews (PROSPERO) with the CRD42022353578 ID, which was then followed throughout the process. The 27-item PRISMA (preferred reporting items for systematic reviews and meta-analyses) statement was used as the reporting model for this systematic review to ensure inclusiveness (22). Furthermore, the 12-item “PRISMA for abstract” extension was used to write the abstract (23).

### Search strategy

A complete search strategy used for each database is reported in **Supplementary Table 1**. Two researchers independently searched until Aug 2022 for relevant articles published (with no restriction of publication year) in the following data sources: PubMed, web of science, and Scopus using the following search terms: “Aminophylline” OR “Theophylline ethylenediamine” OR “Theophylline-ethylenediamine” AND “Topical” OR “Topical administration” OR “Cream” OR “Lotion” OR “Local” OR “Subcutaneous” OR “Fat” OR “Fat burn” OR “Fat burner” OR “Fat burning” OR “Fat reduction” OR “Fat reducer” OR “Fat loss” OR “Lipolysis” OR “Contouring” OR “Body contouring” OR “Waist” OR “Thigh” OR “Skin” OR “Circumference” OR “Regional” OR “Cellulite” OR “Adipose tissue” OR “Adiposity” OR “Obesity” OR “Mesotherapy” OR “Cosmetic”. Studies with any search terms in their title or abstract were found, and their abstracts were pooled and imported into Endnote Reference Manager. After excluding duplicate studies and completing screening stages, the research team decided which studies will finally be chosen.

### Eligibility criteria and study selection

Obesity and cellulite were designated as the study’s exposures of interest. The intervention was defined as topical aminophylline application, and the local fat reduction in the studied body area, as our primary outcome of interest, was compared between exposed and unexposed populations. After excluding duplicate studies, two independent researchers performed the first screening stage, examining the remaining articles in accordance with the study’s stated objectives. Reviews, animal studies, experimental studies, clinical trials, and editorial or other types of letters were excluded at this stage.

Through the second screening stage, clinical trials were included if:

- Aminophylline was used in topical form (cream, lotion, ointment, and gel) alone or in combination with other ingredients.
- Desired information on the participants was provided.
- Results were reported as the amount of subcutaneous fat reduced.

Studies got excluded if:

- The study was on the production process of topical formulation.
- Fat reduction was not reported at all or as a result of using topical aminophylline.

Each researcher reported the studies they had picked after the second screening stage, and the team decided which studies would ultimately be selected for data synthesis. Disagreements were settled, if necessary, by a discussion with a third research team member.

### Data extraction

Data from papers were individually gathered by two reviewers and entered into Microsoft Excel.

The data that was extracted included:

- Publication details: author(s), title, journal, date, stated aims.
- Study characteristics: study location, number of participants, participants’ characteristics, pharmaceutical form of aminophylline, other ingredients of topical formulation (if applicable), and body area(s) studied.
- Critical data: Topical formulation and concentration of active pharmaceuticals, administration routine, other interventions (if applicable), adverse effects (if reported), and final results in as much detail as possible (mean ± SD or median ± SE).

### Risk of bias assessment

Using the Cochrane Collaboration’s approach (24), two reviewers independently categorized studies as having a low, high, or uncertain risk of bias in various domains. Then, if necessary, disagreements were discussed with a third researcher on the research team in order to be resolved.

The risk assessment method developed by Cochrane Collaboration evaluates the probability of bias in six areas for each study. Areas are “Selection bias”, “Performance bias”, “Detection bias”, “Attrition bias “, “Reporting bias”, and “Other bias”. The “selection bias” section checks whether the study clearly describes the allocation sequence generation process so that it can be determined whether or not it should result in comparable groupings. Furthermore, it also verifies whether the study has indicated if intervention allocations could have been anticipated before or during enrollment by providing enough information about the technique employed to disguise the allocation sequence. The “Performance bias” section monitors if the study describes the procedures taken, if any, to prevent trial participants’ intervention status from being known to researchers. The “Detection bias” section ensures if the study describes all procedures taken, if any, to prevent participants’ particular interventions from being known during outcome evaluation. The “Attrition bias” determines whether the study describes how comprehensive the outcome data is for each primary outcome, taking attrition and analytical exclusions into account. And finally, the “Reporting bias” section examines if the study describes the methodology used to analyze selective outcome reporting.

## Results

### Study selection

**Figure 1:**
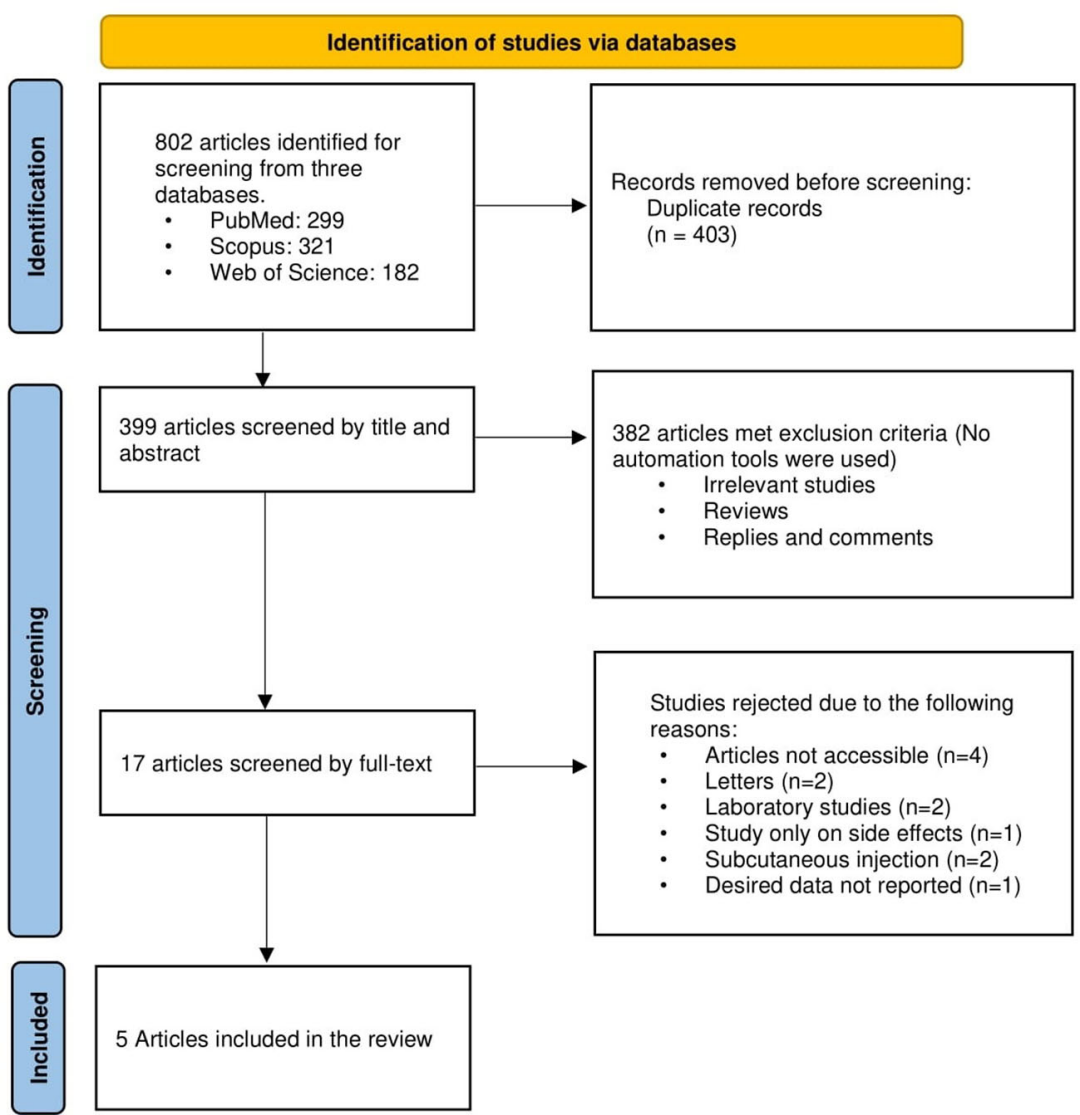
A summary of the study screening process.

**Figure 1** displays a flowchart of the study selection process’ summary. Using the aforementioned search protocol, 802 articles were initially found, including 299 from PubMed, 321 from Scopus, and 182 from Web of Science. The total number of 399 items made it to the first stage of screening after 403 duplicates were eliminated. Then, 382 articles were excluded as a consequence of the first step’s title and abstract screening because they were either unrelated to the issue or did not include the information that was sought. Twelve studies were eliminated from the 17 remaining papers that underwent full-text screening because there was insufficient information presented on the relationship between the topical use of aminophylline and local fat reduction. Finally, data from five papers (17, 18, 25–27) were used after they satisfied all inclusion criteria.

### Basic characteristics of the selected studies

**Table 1** shows the basic characteristics of the five studies that were finally included. All studies except one that was carried out in England (25), were done in the US. Six sub-trials were included in one of the studies (18), of which five were utilized in this study. That one study was dropped because it utilized injection administration. In general, sample sizes in studies were small but varied, ranging from five to 25 participants and totaling 117 people, which were mostly women. The majority of the research was done on participants who were obese, thought they were obese, or thought their thighs were unattractively fat. With the exception of two studies that created lotion (27) and gel (17) forms, all other studies formulated creams and ointments. Three of the trials added additional components to the formulation. Forskolin, yohimbine, caffeine, L-carnitine, and gotu kola (*Centella asiatica*) were added in two of the studies to boost the fat-burning impact, while glycolic acid was added in the other to increase cream penetration into the skin. Three trials used the Aquaphor^®^ base for formulating ointment. Regarding the body area(s) tested, all studies tested on the thighs, except for one study that tested the topical formulation on the waist (26). Among all, two studies examined the buttocks as well (17, 25).

**Table 1:** Basic characteristics of clinical trials evaluating the effect of topical aminophylline in local fat reduction.

Author, year	Study location	Number of participants	Participants’ characteristics	Pharmaceutical form	Other ingredients	Body area(s) studied
Artz and Dinner (17)	United States	12 volunteers	–	Gel	–	Lateral thighs and buttocks
Greenway et al. (18) (Trial 2)	United states	5 women	Participants were more than 20% above their desirable weights.	Ointment	• Aquaphor^®^ base • Forskolin • Yohimbine	Thighs
Greenway et al. (18) (Trial 3-3)	United states	5 women	Participants were greater than 20% overweight.	Ointment	Aquaphor^®^ base	Thighs
Greenway et al. (18) (Trial 4)	United states	23 women	Participants were greater than 20% overweight.	Ointment	Aquaphor^®^ base	Thighs
Greenway et al. (18) (Trial 5)	United states	11 women	• All participants felt that their thighs were undesirably fat. • Some women wanted to lose weight while others were satisfied with their present weight.	Cream	–	Thighs
Greenway et al. (18) (Trial 6)	United states	12 women	• All participants felt that their thighs were undesirably fat. • Some women wanted to lose weight while others were satisfied with their present weight.	Cream	–	Thighs
Collis et al. (25)	England	17 women	Participants were over 18 years old with cellulite of the thighs and buttocks.	Cream	10% glycolic acid	Thighs and buttocks
Caruso et al., (26)	United states	25 men and women	• All participants were obese and overweight with a BMI greater than 27 kg/m^2^. • Participants’ age was between 21 and 65 years. • Participants had android fat distribution (characterized by a waist to hip ratio >0.80 in women or >1.0 in men).	Cream	–	Waist
Escalante et al. (27)	United states	7 women	–	Lotion	• Caffeine, • Yohimbe • L-Carnitine • Gotu kola	Thighs

### Outcomes

A detailed summary of studies evaluating the effect of topical aminophylline in local fat reduction is provided in **Table 2**. As can be seen, several concentrations of aminophylline were used in different studies. Among all, the highest concentration of aminophylline used was 10%, with the administration of five times a week on participants’ one thigh (18). The other thigh was considered to be the control for comparing the fat reduction amount. Although participants were suggested to stick to a 900 to 1,100 kcal/day diet, they were not suggested to do any exercises. After six weeks, there had been a higher loss in participants’ thigh girth in the treated thigh than in the control thigh, with a mean ± SEM difference of 0.77 ± 0.66 cm for the lower girth and 0.78 ± 0.89 cm for the upper girth (p < 0.001). Heart rate, blood pressure, and blood chemistry all remained unchanged. Additionally, no theophylline could ever be found in blood samples, and no allergic reactions were documented.

**Table 2:** A summary of studies evaluating the effect of topical aminophylline in local fat reduction.

Author, year	Topical formulation	Administration	Other interventions	Results	Adverse effects
Artz and Dinner (17)	2% aminophylline	Twice a day for 12 weeks	• Patients avoided any medications affecting theophylline serum levels.	The thigh circumference was decreased an average of 0.5 cm, but with no weight reduction.	• Total cholesterol, triglycerides, HDL, and LDL did not show any significant changes after three months. • Aminophylline serum levels did not raise.
Greenway et al. (18) (Trial 2)	1.2×10^-5^ M forskolin + 2.5×10^-4^ M yohimbine + 1.3×10^-2^ M aminophylline	Five times a week for four weeks	• 600 kcal/day diet • Patients were encouraged to follow a walking program • To increase transcutaneous absorption, the thighs were wrapped in warm 600 to 900 mOsm/L magnesium sulphate solutions for 30 minutes before each of the ointment applications. • An occlusive plastic wrap was placed over the area to which the ointment was applied throughout the 4-week study period.	All participants lost more girth on the treated thigh than the control thigh with a mean ± SEM difference of 2.03 ± 1.36 cm (p<0.05)	• One of the ladies who participated in this study, which was carried out in the summer, experienced a heat rash under the occlusive plastic wrap on both legs that went away after the plastic wrap was removed.
Greenway et al. (18) (Trial 3-3)	1.3×10^-2^ M aminophylline	Five times a week for four weeks	• 800 kcal/day diet • Patients were encouraged to follow a walking program • To increase transcutaneous absorption, the thighs were wrapped in warm 600 to 900 mOsm/L magnesium sulphate solutions for 30 minutes before each of the ointment applications.	All participants lost more girth on the treated thigh than the control thigh with a mean ± SEM difference of 1.5 ± 0.77 cm (p<0.02)	• There were no rashes or other adverse events. • There were no changes in blood pressure or pulse
Greenway et al. (18) (Trial 4)	10% aminophylline	Five times a week for six weeks	• 900 to 1,100 kcal/day diet • No specific exercise	Thigh girth loss was greater in the treated than in the control thigh at the end of the study with a mean ± SEM difference of 0.77 ± 0.66 cm for the lower girth and 0.78 ± 0.89 cm for the upper girth (p<0.001)	• No significant changes were seen in the chemistry results. • No theophylline could be detected at any time point. • Patch testing showed no allergy. • There were no significant changes in pulse rate or blood pressure.
Greenway et al. (18) (Trial 5)	2% aminophylline	Five times a week for six weeks	• No specific diet • No specific exercise	Participants lost more girth from the treated than from the control thigh with a mean ± SEM difference of 1.21 ± 0.31 cm (p<0.01)	• There was no skin irritation with patch testing. • Three weeks after the start of the study, one woman developed a rash on the leg being treated with active cream. The cream was stopped and the rash resolved • Theophylline levels were undetectable
Greenway et al. (18) (Trial 6)	0.5% aminophylline	Five times a week for five weeks	• No specific diet • No specific exercise	All 12 participants lost more girth on the treated thigh than the control thigh at 5 weeks of treatment with a mean ± SEM difference of 3.08 ± 0.27 cm (p<0.0011)	• The chemistry panel showed significant decreases in ALT, LDH, globulin and creatinine, but these were felt to be clinically insignificant since the changes were within the normal range for the test. • There were no rashes. • Patch testing was negative. • Theophylline levels were below the detectable threshold.
Collis et al. (25)	2% aminophylline	Twice a day for 12 weeks	• Patients were instructed to maintain their lifestyle	There was no significant difference (p=0.4 to 0.9) in the fat depth and thigh girth between treatment and control groups.	• Nine out of 35 patients developed a dermatologic reaction.
Caruso et al., (26)	0.5% aminophylline	Twice a day for 12 weeks	• 1200 kcal/day diet • Patients were encouraged to follow a walking program	The reduction in waist circumference was 11 ± 1.0 cm in the aminophylline cream group and 5.0 ± 0.6 cm in the control group (p < 0.001).	• All monthly aminophylline levels were undetectable. • There were no adverse events • There were no allergic reactions to the cream. • Blood pressure and pulse remained in the normal range throughout the study.
Escalante et al. (27)	Aminophylline, caffeine, yohimbe, l‐carnitine, and gotu kola	Twice a day for four weeks	• 1520 ± 321 kcal/day diet • Patients were encouraged to follow a walking program	Reduction in thigh circumference was 1.2 cm compared to 0.8 cm in control groups.	–

In the case of 2% aminophylline concentration, there were three experiments. None of the three studies’ participants maintained a regular diet or exercise routine. In the first two studies (17, 25), using twice-daily treatment, one of them indicated a mean 0.5 cm reduction in thigh fat after 12 weeks, and the other found no significant difference in the fat reduction after 12 weeks. The third study (18), applying the cream five times a week, reported that participants lost more girth from the treated than from the control thigh with a mean ± SEM difference of 1.21 ± 0.31 cm (p < 0.01) after six weeks. While one of the studies reported no side effects at all, two other studies reported skin rashes in nine out of 35, and one out of 11 participants.

Regarding 0.5% aminophylline concentration, there existed two experiments. In one of them (18), participants applied the topical formulation five times per week for five weeks without engaging in any physical activity or dietary changes. As a result, the treated thigh lost more circumference than the control thigh, with a mean ± SEM difference of 3.08 ± 0.27 cm (p < 0.001) in all 12 participants. Neither any dermatologic rashes nor any theophylline serum levels were detected. Moreover, although alanine transaminase (ALT), lactate dehydrogenase (LDH), globulin, and creatinine levels in the chemistry panel all significantly decreased, the changes were deemed to be clinically unimportant because they were within the test’s normal range. In the second research on the 5% aminophylline concentration (26), the topical formulation was administered twice daily on the participants’ waist, the subjects consumed 1200 kcal per day, and walking programs were suggested. After 12 weeks, the mean reduction in waist circumference was 11 ± 1.0 cm in the aminophylline cream group and 5.0 ± 0.6 cm in the control group (p < 0.001). Blood pressure and heart rate remained unchanged throughout the study, no allergic reactions were reported, and all monthly aminophylline levels were undetectable.

There existed one study using 1.3×10^-2^ M aminophylline concentration in the topical form five times a week (18). Participants were encouraged to stick to a 800 kcal/day diet and to follow a walking program. For 30 minutes before each application of ointment, the thighs were wrapped in heated 600 to 900 mOsm/L magnesium sulphate solutions to increase absorption. After four weeks, on the treated thigh, more girth was decreased than on the untreated thigh with a mean ± SEM difference of 1.5 ± 0.77 cm (p <0.02). Regarding side effects, no rashes and no changes in blood pressure and heart rate were reported.

Finally, two studies added other active ingredients to their topical formulation. In the first one (18), the formulation was 1.2×10^-5^ M forskolin plus 2.5×10^-4^ M yohimbine plus 1.3×10^-2^ M aminophylline and was administered five times a week. Participants were encouraged to stick to a 600 kcal/day diet and follow a walking program. To increase transcutaneous absorption, the thighs were wrapped in warm 600 to 900 mOsm/L magnesium sulphate solutions for 30 minutes before each of the ointment applications. Moreover, the region to which the ointment was applied was covered with an occlusive plastic wrap. After four weeks, the treated thigh of each subject shrunk more than the control thigh with a mean ± SEM difference of 2.03 ± 1.36 cm (p <0.05). In the case of side effects, one of the women who took part in this trial, which was conducted in the summer, developed a heat rash on both legs under the occlusive plastic wrap, which disappeared once the plastic wrap was taken off. Another study (27), which supplemented the topical formulation with other active components, combined the effects of aminophylline, caffeine, yohimbe, l-carnitine, and gotu kola to reduce localized fat. The formulation was used twice a day along with a 1520 ± 321 kcal/day diet and walking program. After four weeks, the reduction in thigh circumference was 1.2 cm compared to 0.8 cm in the control groups.

### Risk of bias assessment

**Supplementary Table 2** shows the results of Cochrane Collaboration’s tool for assessing the risk of bias in the final retrieved studies. As can be observed, in the “Random sequence generation” area all research had an uncertain risk of bias, except for one study (27) that had a low risk of bias. The results in the “Allocation concealment” domain were exactly the same. In the “Blinding of participants and personnel” area, all research had a low risk of bias, except for one study that had a high risk of bias (17). Results in the “Blinding of outcome assessment” section were varied, with two, one, and two studies having high, unclear, and low risk of bias, respectively. Regarding the “Incomplete outcome data” domain, studies had one unclear, three lows, and one high risk of bias. In the case of the “Selective reporting” area, all studies had a low risk of bias. Finally, no studies had other types of biases.

## Discussion

This research established the possibility for topical formulations with aminophylline as their active component to be employed as local fat burners. Furthermore, even though it was demonstrated that these topical treatments alone can reduce subcutaneous fat, it seems that adding even a slight exercise and diet may increase their effectiveness.

Aminophylline is a bronchodilator agent that is FDA-approved for managing acute asthma and is available in the form of oral capsules, oral tablets, and intravenous solutions. Although it is not FDA-approved for local fat reduction, some aminophylline-containing creams are currently available in the store and are advertised as cellulite removers. Whether or not they are obese, many women worry about how their thighs, buttocks, waist, and double chin look. Women who turn to surgical treatments as a kind of therapy because they are so upset with the way their body fat looks benefit from topical forms of fat burning the most. Topical lipolysis is less dangerous, with avoiding the associated dangers of surgery, such as scarring and infection hazards, as well as anesthesia risks. Moreover, most of the ladies who participated in trials evaluating the potency of topical aminophylline in local fat reduction reported having a better sense of how their body looked, which may have actually enhanced how they felt about themselves. However, although the topical formulation of aminophylline seemed to be helpful in reducing localized fat, trials that did not take into account diets and exercise routines for participants did not report many cases of weight loss. Therefore, the difference in subcutaneous fat and lack of difference in weight reduction between the two groups point to a superficial alteration in fat distribution.

### Side effects

Regarding side effects, all trials that tracked participant blood chemistry, blood pressure, blood levels of aminophylline and theophylline, and pulse rate found no significant changes, therefore it appears that this formulation is safe in terms of adverse effects. The only unfavorable consequence that was noted occasionally was skin rashes. Regarding why rashes happened, some studies suggested possible mechanisms. It was recognized that aminophylline, which is made up of two theophylline molecules linked by an ethylenediamine molecule, is a skin irritant (26). In studies using a simple base cream, it was reported that a chemical reaction with aminophylline causes a common cream base to become yellow. This yellow cream fails to work and gave some test subjects rashes (26). As a result, it was proven that utilizing a specially created cream base that stabilizes the aminophylline decreases skin rashes in addition to assisting in adjusting its skin penetration.

### Possible mechanism of action

Xanthenes are thought to increase collagen production, lipolysis, microcirculation, and thermogenesis along with decreasing adipogenesis (28–31). In the case of their lipolytic actions, publications have shown that beta-adrenergic stimulation directly increases the cyclic adenosine monophosphate (cAMP) concentrations of fat cells in adipocytes, which is believed to be the underlying mechanism (28, 30, 32, 33). The increased cAMP levels cause protein kinase A to phosphorylate the activating hormone-sensitive lipase (HSL). Now, triglycerides are hydrolyzed by phosphorylated HSL into free fatty acids, glycerol, diglycerides, and monoglycerides (34). Parallelly, since xanthines are thought to inhibit phosphodiesterase (PDE), an enzyme involved in the degradation of cAMP, as well, the inhibition of PDE further raises cAMP activity. Therefore, the researchers believe that by applying aminophylline topically to suppress PDE, local cAMP concentrations and local lipolysis would both rise (28, 30, 35–37).

### Strength and limitations

This study was one of the first systematic reviews about local fat burning by topical formulations. To ensure comprehensiveness, we reported the outcomes of studies using various aminophylline concentrations in their topical formulations, as well as formulations using substances other than aminophylline alone. However, there are some limitations to this study. First of all, despite a comprehensive search strategy, there were few recent research on our subject, and the sources used for this study are relatively old. One possible reason for this oldness may be that most of the studies concentrate on the intravenous administration of aminophylline, and the topical use is not well known. Second, there isn’t enough information accessible for each concentration to allow for useful comparison because there aren’t many references, and each reference employed different concentrations. Third, some references exhibited a high risk of bias in particular areas after utilizing the Cochrane Collaboration’s methodology to assess the risk of bias.

## Conclusion

Aminophylline topical formulation offers a safe, effective, and much less invasive alternative to cosmetic surgery for the localized reduction of fat. Conducting further trials to compare the efficacy of different concentration of aminophylline is suggested for future works, as well as evaluating the efficacy of other xanthines in local fat burning.

## Data availability statement

The original contributions presented in the study are included in the article/**Supplementary Material**. Further inquiries can be directed to the corresponding author.

## Author contributions

RA and AH: Writing the manuscript, Collecting the data. ED and MQ: Designing the study, Checking and revising, Supervision. All authors contributed to the article and approved the submitted version.
