# Supplementary material for: The efficacy of topical aminophylline in local fat reduction: A systematic review
Source: Front Endocrinol (Lausanne). 2023 Feb 16;14:1087614. doi: 10.3389/fendo.2023.1087614 (PMC9978326; doi:10.3389/fendo.2023.1087614)
Supplement: Supplementary file 1 [file Table_1.docx]

**Supplementary table 1**. The syntax used for searching in each database.

| **Database** | **Syntax** |
| --- | --- |
| **PubMed** | (Aminophylline[tiab] OR “theophylline ethylenediamine” OR “theophylline-ethylenediamine”) AND (“fat” OR “fat burn” OR “fat burner” OR “fat burning” OR “fat reduction” OR “fat reducer” OR “fat loss” OR “lipolysis” OR “contouring” OR “body contouring” OR “waist” OR “thigh” OR “skin” OR “circumference” OR “regional” OR “cellulite” OR “adipose tissue” OR “adipose” OR “adiposity” OR “obesity” OR “mesotherapy” OR “cosmetic” OR topical OR “topical administration” OR “cream” OR “lotion” OR “local” OR “subcutaneous”) |
| **Web of Science** | (TS=(aminophylline) OR ALL=(“theophylline ethylenediamine”) OR ALL=(“theophylline-ethylenediamine”)) AND (ALL=(fat) OR ALL=(“fat burn”) OR ALL=(“fat burner”) OR ALL=(“fat burning”) OR ALL=(“fat reduction”) OR ALL=(“fat reducer”) OR ALL=(“fat loss”) OR ALL=(“lipolysis”) OR ALL=(“contouring”) OR ALL=(“body contouring”) OR ALL=(“waist”) OR ALL=(“thigh”) OR ALL=(“skin”) OR ALL=(“circumference”) OR ALL=(“regional”) OR ALL=(“cellulite”) OR ALL=(“adipose tissue”) OR ALL=(“adipose”) OR ALL=(“adiposity”) OR ALL=(“obesity”) OR ALL=(“mesotherapy”) OR ALL=(“cosmetic”) OR ALL=(“topical”) OR ALL=(“topical administration”) OR ALL=(“cream”) OR ALL=(“lotion”) OR ALL=(“local”) OR ALL=(“subcutaneous”)) |
| **Scopus** | ( TITLE-ABS ( aminophylline )) AND ( TITLE-ABS ( fat ) OR TITLE-ABS ( "fat burn" ) OR TITLE-ABS ( "fat burner" ) OR TITLE-ABS ( "fat burning" ) OR TITLE-ABS ( "fat reduction" ) OR TITLE-ABS ( "fat reducer" ) OR TITLE-ABS ( "fat loss" ) OR TITLE-ABS ( "lipolysis" ) OR TITLE-ABS ( "contouring" ) OR TITLE-ABS ( "body contouring" ) OR TITLE-ABS ( "waist" ) OR TITLE-ABS ( "thigh" ) OR TITLE-ABS ( "skin" ) OR TITLE-ABS ( "circumference" ) OR TITLE-ABS ( "regional" ) OR TITLE-ABS ( "cellulite" ) OR TITLE-ABS ( "adipose tissue" ) OR TITLE-ABS ( "adipose" ) OR TITLE-ABS ( "adiposity" ) OR TITLE-ABS ( "obesity" ) OR TITLE-ABS ( "mesotherapy" ) OR TITLE-ABS ( "cosmetic" ) OR TITLE-ABS ( "topical" ) OR TITLE-ABS ( "topical administration" ) OR TITLE-ABS ( "cream" ) OR TITLE-ABS ( "lotion" ) OR TITLE-ABS ( "local" ) OR TITLE-ABS ( "subcutaneous" ) ) |
