# Supplementary material for: The efficacy of topical aminophylline in local fat reduction: A systematic review
Source: Front Endocrinol (Lausanne). 2023 Feb 16;14:1087614. doi: 10.3389/fendo.2023.1087614 (PMC9978326; doi:10.3389/fendo.2023.1087614)
Supplement: Supplementary file 2 [file Table_2.docx]

**Supplementary table 2.** Cochrane Collaboration’s tool for assessing risk of bias in the final retrieved studies

| **Risk of bias** | **Source of bias** | **Bias domain** | **Author, year** |
| --- | --- | --- | --- |
| Unclear | Random sequence generation | Selection bias | Artz & Dinner, 1995 |
| Unclear | Allocation concealment |  |  |
| High risk | Blinding of participants and personnel | Performance bias |  |
| High risk | Blinding of outcome assessment | Detection bias |  |
| Unclear | Incomplete outcome data | Attrition bias |  |
| Low risk | Selective reporting | Reporting bias |  |
| - | Anything else, ideally prespecified | Other bias |  |
| Unclear | Random sequence generation | Selection bias | Greenway et al., 1995 |
| Unclear | Allocation concealment |  |  |
| Low risk | Blinding of participants and personnel | Performance bias |  |
| Unclear | Blinding of outcome assessment | Detection bias |  |
| Low risk | Incomplete outcome data | Attrition bias |  |
| Low risk | Selective reporting | Reporting bias |  |
| - | Anything else, ideally prespecified | Other bias |  |
| Unclear | Random sequence generation | Selection bias | Collis et al., 1999 |
| Unclear | Allocation concealment |  |  |
| Low risk | Blinding of participants and personnel | Performance bias |  |
| High risk | Blinding of outcome assessment | Detection bias |  |
| Low risk | Incomplete outcome data | Attrition bias |  |
| Low risk | Selective reporting | Reporting bias |  |
| - | Anything else, ideally prespecified | Other bias |  |
| Unclear | Random sequence generation | Selection bias | Caruso et al., 2006 |
| Unclear | Allocation concealment |  |  |
| Low risk | Blinding of participants and personnel | Performance bias |  |
| Low risk | Blinding of outcome assessment | Detection bias |  |
| Low risk | Incomplete outcome data | Attrition bias |  |
| Low risk | Selective reporting | Reporting bias |  |
| - | Anything else, ideally prespecified | Other bias |  |
| Low risk | Random sequence generation | Selection bias | Escalante et al., 2019 |
| Unclear | Allocation concealment |  |  |
| Low risk | Blinding of participants and personnel | Performance bias |  |
| Low risk | Blinding of outcome assessment | Detection bias |  |
| High risk | Incomplete outcome data | Attrition bias |  |
| Low risk | Selective reporting | Reporting bias |  |
| - | Anything else, ideally prespecified | Other bias |  |
